# The Molecular Priming of Defense Responses is Differently Regulated in Grapevine Genotypes Following Elicitor Application against Powdery Mildew

**DOI:** 10.3390/ijms21186776

**Published:** 2020-09-15

**Authors:** Chiara Pagliarani, Amedeo Moine, Walter Chitarra, Giovanna Roberta Meloni, Simona Abbà, Luca Nerva, Massimo Pugliese, Maria Lodovica Gullino, Giorgio Gambino

**Affiliations:** 1 Institute for Sustainable Plant Protection, National Research Council (IPSP-CNR), Strada delle Cacce 73, 10135 Torino, Italy; amedeo.moine@ipsp.cnr.it (A.M.); walter.chitarra@crea.gov.it (W.C.); simona.abba@ipsp.cnr.it (S.A.); luca.nerva@crea.gov.it (L.N.); giorgio.gambino@ipsp.cnr.it (G.G.); 2 Research Centre for Viticulture and Enology, Council for Agricultural Research and Economics (CREA-VE), Via XXVIII Aprile 26, 31015 Conegliano, Italy; 3 Centre of Competence for the Innovation in the Agro-Environmental Sector (AGROINNOVA), University of Torino, Largo Paolo Braccini 2, 10095 Grugliasco (TO), Italy; giovannaroberta.meloni@unito.it (G.R.M.); massimo.pugliese@unito.it (M.P.); marialodovica.gullino@unito.it (M.L.G.); 4 Department of Agricultural, Forest and Food Sciences (DISAFA), University of Torino, Largo Paolo Braccini 2, 10095 Grugliasco (TO), Italy

**Keywords:** resistance inducers, *Vitis vinifera*, *Erysiphe necator*, gene expression reprogramming, RNAseq, secondary metabolites, hormone signaling

## Abstract

Molecular changes associated with response to powdery mildew (PM) caused by *Erysiphe necator* have been largely explored in *Vitis vinifera* cultivars, but little is known on transcriptional and metabolic modifications following application of resistance elicitors against this disease. In this study, the whole transcriptome sequencing, and hormone and metabolite analyses were combined to dissect long-term defense mechanisms induced by molecular reprogramming events in PM-infected ‘Moscato’ and ‘Nebbiolo’ leaves treated with three resistance inducers: acibenzolar-S-methyl, potassium phosphonate, and laminarin. Although all compounds were effective in counteracting the disease, acibenzolar-S-methyl caused the most intense transcriptional modifications in both cultivars. These involved a strong down-regulation of photosynthesis and energy metabolism and changes in carbohydrate accumulation and partitioning that most likely shifted the plant growth-defense trade-off towards the establishment of disease resistance processes. It was also shown that genotype-associated metabolic signals significantly affected the cultivar defense machinery. Indeed, ‘Nebbiolo’ and ‘Moscato’ built up different defense strategies, often enhanced by the application of a specific elicitor, which resulted in either reinforcement of early defense mechanisms (e.g., epicuticular wax deposition and overexpression of pathogenesis-related genes in ‘Nebbiolo’), or accumulation of endogenous hormones and antimicrobial compounds (e.g., high content of abscisic acid, jasmonic acid, and viniferin in ‘Moscato’).

## 1. Introduction

Climate change has been seriously impacting the productivity of agricultural ecosystems in the world [1,2]. In southern Europe, including Italy, the frequency and duration of drought events and heat waves dramatically increased in the last years, breaking new records in terms of temperature and/or precipitation anomalies [3] and causing the intensification of pest and pathogen spread, with devastating effect on the crop yield potential [4,5].

Grapevine (*Vitis vinifera* L.) is one of the most economically important fruit crops, worldwide renowned for winemaking and grape consumption. Cultivars of *V. vinifera* are heavily impacted by environmental variables [6], as they are cultivated in vineyard areas that span diverse climates across the planet [7]. During the grape pre-and post-harvest phases, infections due to fungi such as *Erysiphe necator*, the causal agent of powdery mildew (PM) [8,9], represent, from an economic perspective, some of the most damaging threats for grape growers. During the last years, attacks due to powdery mildews have been seriously worsened by climate alterations, as they are highly favored by increases in CO_2_ and temperature [10,11]. Indeed, the enhancing effect of increased CO_2_ concentrations and high temperatures on powdery mildew was experimentally demonstrated in grapevine plants grown within phytotrons [12].

A serious drawback of the intensification of fungal diseases due to climate alterations is the massive use of chemical fungicides in most of the European countries [13], which has inevitably led to environmental sustainability issues [14,15] and fungicide resistance [16,17]. The necessity of progressively reducing chemical treatments, as established by the European Commission (EC) legislation (Directive 2009/128/EC), is thus making challenging the availability of new plant protection strategies. Advances to address this question have been provided in the breeding sector through dedicated programs of marker-assisted selection that led to the production of hybrid *Vitis* genotypes harboring resistance to PM [18,19]. In addition to conventional breeding approaches, modern genome editing techniques represent a promising strategy to ensure increased cultivar resilience and productivity [20]. Nevertheless, the exploitation of genomic technologies in viticulture, and more in general in the agricultural sector, is still prevented in the EU, and encounters restrictions in several other countries [21]. Additionally, the commercialization of resistant grapevine hybrids is not free of issues related to the preservation of the geographical identity of grape cultivars and the consumer’s appreciation of wine-derived products [22].

The application of environmentally friendly products in substitution of chemical compounds is therefore the most suitable action to take now [23]. Encouraging results in this field have been achieved using bioactive elements, natural-derived products and salts, such as silicon and, recently, laminarin and potassium phosphonates. These substances function as resistance elicitors by stimulating endogenous plant defense responses and/or inhibiting the fungus colonization and proliferation [24,25,26,27,28]. Other promising outcomes have been obtained using the acibenzolar-S-methyl [29], a benzothiadiazole analog to salicylic acid (SA) suggested to induce systemic acquired resistance (SAR) by mimicking host-pathogen reactions [30].

Despite the mentioned efforts, the successful application of these novel fungicides as well as their registration among the products admitted in viticulture strictly need a thorough understanding of their biological activity [31,32] and also rely on comprehensive knowledge of the plant machinery controlling defense responses to fungal pathogens. Insights into resistance-involved molecular pathways have been provided by applying “omics” technologies, like microarray [33,34,35] and high throughput sequencing [36]. However, gaps remain in the study of molecular triggers that tune the interaction between grapevine and fungal pathogens [9]. Particularly, further efforts should be made to deepen our knowledge about networks of immunity mediators (e.g., involving hormonal and secondary metabolic signaling) leading to either cultivar susceptibility or resistance [37,38]. To the best of our knowledge, with the exception of a few efforts [39], no studies have been performed at the “omic” level to unveil the molecular signature associated with the reprogramming of defense responses in grapevine plants treated with different resistance inducers against fungal diseases.

The effectiveness of three resistance inducers (acibenzolar-S-methyl (AcS-Mt), laminarin (Lam) and potassium phosphonate (K-Pho)) was recently tested against PM on two *V. vinifera* cultivars, Nebbiolo and Moscato, and their specificity was also assessed by tracking changes in the fungal microbial community-inhabiting the leaf surface [40]. The impact of the same active ingredients on microbial ecology and grape quality was also investigated in a field trial on ‘Nebbiolo’, proving that the yield and vigor of vines were not influenced by the treatments, nor were the production of primary and secondary metabolites [41].

Interestingly, a role for the genotype in influencing both the plant susceptibility to the pathogen and its response to the elicitor application was suggested by [40], based on the observation that disease severity was significantly lower in ‘Moscato’ than ‘Nebbiolo’. In the search for experimental evidence supporting this hypothesis, in the present work, the molecular reprogramming events that characterize distinct defense responses in PM-infected ‘Moscato’ and ‘Nebbiolo’ plants subjected to the same elicitor treatments were investigated. To this aim, whole transcriptional modifications were explored on the same leaves of the previously published research [40] by adopting an RNAseq approach integrated with the analysis of candidate genes and metabolites. Molecular changes associated with treatment- and/or cultivar-specific defense mechanisms were then determined and discussed taking into account alterations in the content of secondary metabolites and hormones.

## 2. Results

**Figure 1:**
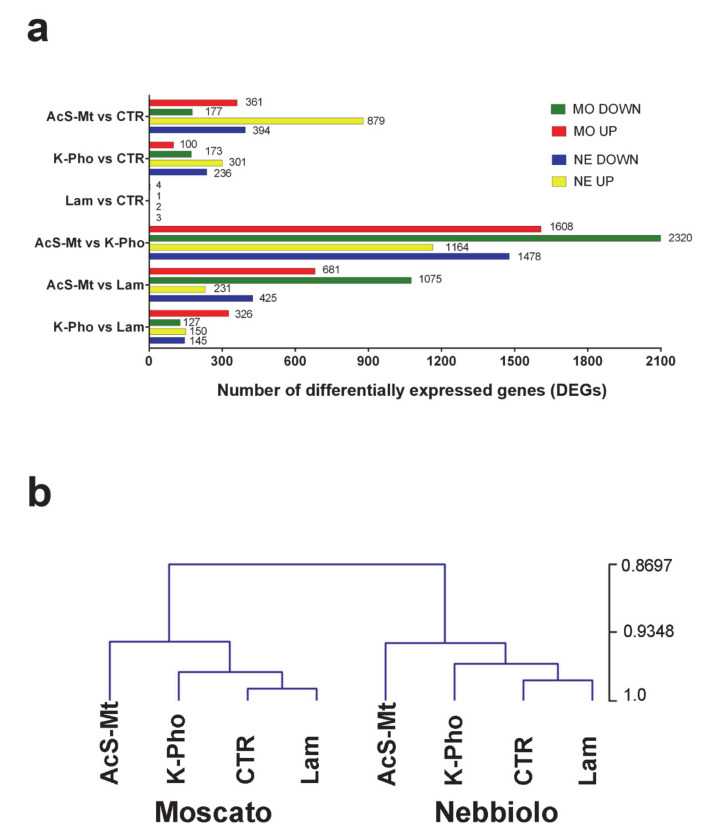
Transcriptome analysis of leaf samples obtained from ‘Nebbiolo’ and ‘Moscato’ plants artificially inoculated with *Erysiphe necator* and then untreated (CTR) or treated with acibenzolar-S-methyl (AcS-Mt), potassium phosphonate (K-Pho), and laminarin (Lam). In (**a**), the total number of differentially expressed genes (DEGs), up (UP) or down (DOWN)-regulated, is shown for each RNAseq comparison beside the bar charts. MO = ‘Moscato’; NE = ‘Nebbiolo’. In (**b**), the dendrogram displays the output of the HCL (Hierarchical Clustering) analysis performed on RNAseq data. The numbers reported next to each node are bootstrap values from 1000 replicates.

The high-throughput sequencing analysis produced an average of 44 million reads per sample that were aligned to the V1 version of the grapevine transcriptome (Table S1). In the preliminary analysis, 6082 out of the 29,970 annotated genes were significantly differentially expressed in ‘Moscato’ and ‘Nebbiolo’ in at least one treatment in all possible combinations of pairwise comparisons (AcS-Mt vs. CTR, K-Pho vs. CTR, Lam vs. CTR, AcS-Mt vs. K-Pho, AcS-Mt vs. Lam, K-Pho vs. Lam) (*p*-value adjusted with Benjamin-Hochberg ≤0.5%; Figure 1a, Table S2). Interestingly, in comparison with CTR, Lam was the treatment that, independently of the genotype, modified the leaf transcriptome the least, showing only five genes modulated in each cultivar (Figure 1a). This finding showed that, although Lam was successful in containing PM attack [40], the overall transcriptomic changes induced by this eco-friendly compound were poor. Conversely, the application of AcS-Mt induced an extensive transcriptional reprogramming, involving a total of 538 significantly differentially expressed genes (DEGs) in ‘Moscato’ and 1273 DEGs in ‘Nebbiolo’, as highlighted in the comparison with CTR (Figure 1a). Similarly, when treatments were compared with each other, AcS-Mt-treated leaves displayed the highest number of DEGs in comparison with the transcriptome of both Lam and K-Pho-treated samples (Figure 1a).

To inspect the relationships of similarity among treatments and CTR, a hierarchical clustering (HCL) analysis was carried out, using as input the whole set of DEGs (Figure 1b). Accordingly, HCL results confirmed that AcS-Mt was the treatment that stimulated the highest rate of transcriptome modulation in both cultivars, while Lam was the closest one to CTR (Figure 1b). The outcome of HCL analysis also revealed a significant distinction between ‘Moscato’ and ‘Nebbiolo’, which formed two separate clades (Figure 1b).

**Figure 2:**
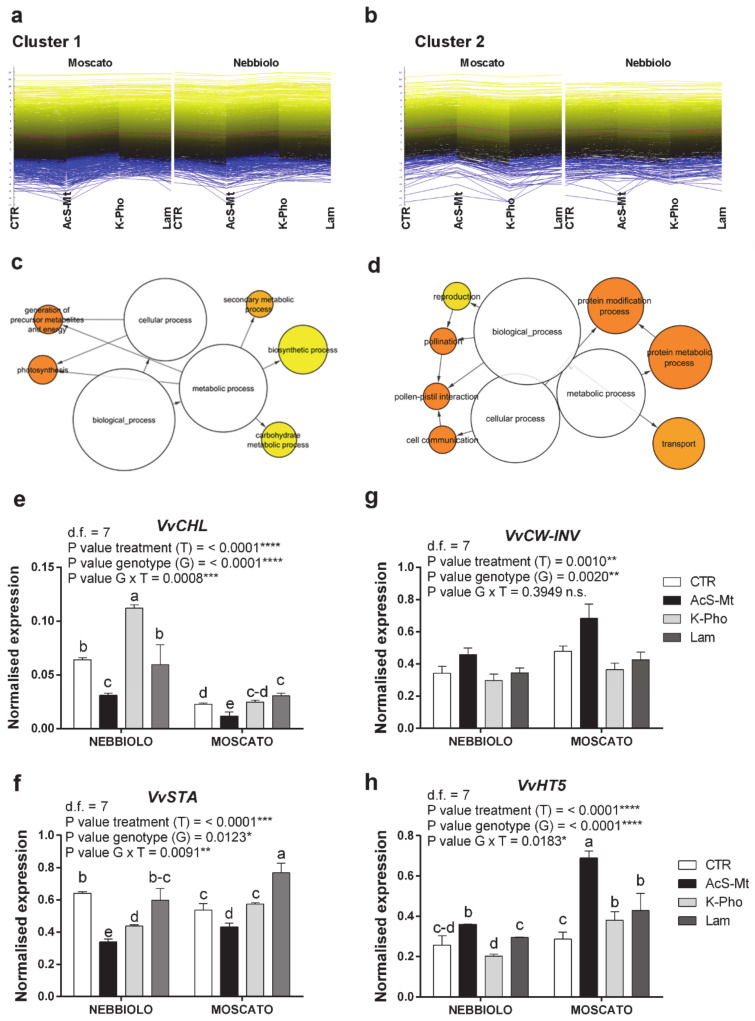
Treatment effect on transcriptional reprogramming of defense responses. (**a**,**b**) Output of HCL (Hierarchical Clustering) analysis conducted on RNAseq data. Clusters of differentially expressed genes (DEGs) showing a down-regulation (**a**, cluster 1) or up-regulation (**b**, cluster 2) transcriptional pattern in association with acibenzolar-S-methyl application (AcS-Mt) in ‘Moscato’ and ‘Nebbiolo’ leaf transcriptomes. (**c**,**d**) Significant enriched GO biological functional categories identified for each group of genes belonging to (**c**) cluster 1 and (**d**) cluster 2 using Cytoscape with the BINGO plug-in and listed according to their enrichment *p*-value (*p* < 0.05). (**e**–**h**) Results of candidate gene expression analysis performed by RT-qPCR assay. Expression profiles of key genes belonging to cluster 1 (**e**-*VvCHL*, VIT_07s0151g00110; f-*VvSTA*, VIT_02s0025g02790) and cluster 2 (**g**-*VvCW-INV*, VIT_09s0002g02320; **h**-*VvHT5*, VIT_05s0020g03140) analyzed in leaf samples taken from ‘Nebbiolo’ and ‘Moscato’ plants artificially inoculated with *Erysiphe necator* and then untreated (CTR) or treated with acibenzolar-S-methyl (AcS-Mt), potassium phosphonate (K-Pho), and laminarin (Lam). Ubiquitin (*VvUBI*) and Actin (*VvACT1*) genes were both used as endogenous controls for the normalization of transcript levels. Significance of genotype, treatment, and genotype × treatment (G × T) interaction was assessed by Tukey’s HSD test for *p* ≤ 0.05 (*), *p* ≤ 0.01 (**), *p* ≤ 0.001 (***), and *p* ≤ 0.0001 (****) and the corresponding results are given above each graph in the figure panel; n.s. = not significant. Lower case letters above bars are reported when the G × T interaction and/or genotype (G) main effects are statistically significant as attested by Tukey’s HSD or Student’s *t*-test, respectively. Error bars represent SE. Three independent biological replicates with three technical replicates each were used for the analysis.

**Figure 3:**
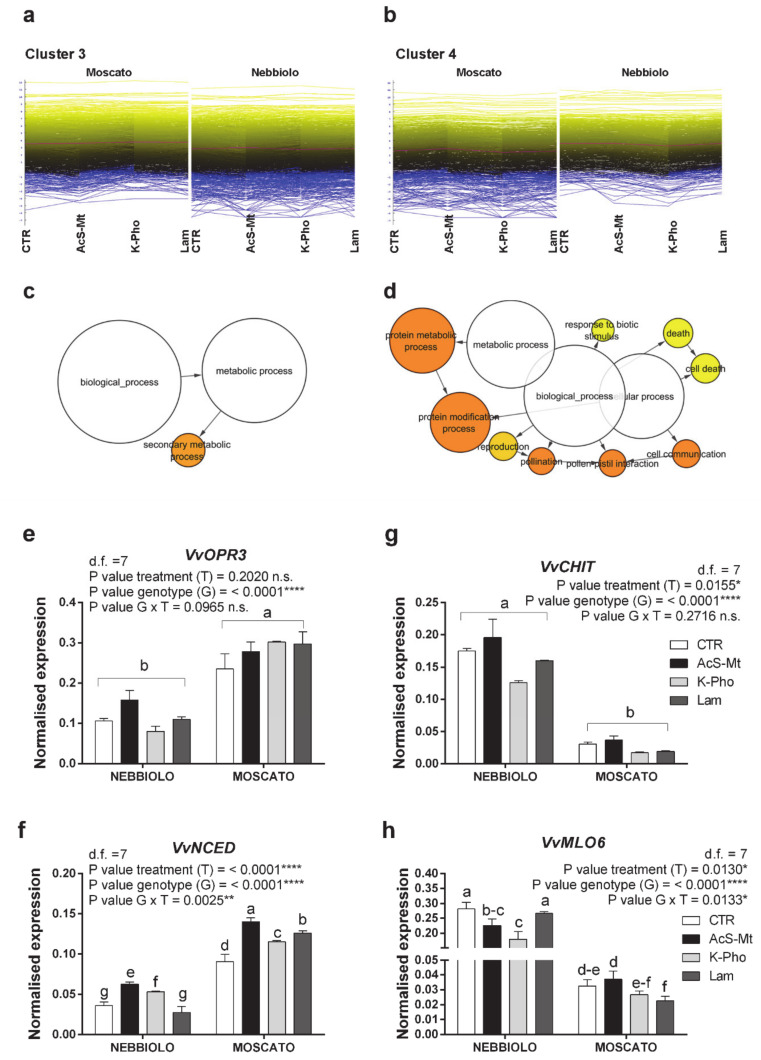
Genotype effect on transcriptional reprogramming of defense responses. (**a**,**b**) Output of HCL (Hierarchical Clustering) analysis conducted on RNAseq data. Clusters of differentially expressed genes (DEGs) mainly upregulated in ‘Moscato’ (**a**, cluster 3) or ‘Nebbiolo’ (**b**, cluster 4) leaf transcriptomes. (**c**,**d**) Significant enriched GO biological functional categories identified for each group of genes belonging to (**c**) cluster 3 and (**d**) cluster 4 using Cytoscape with the BINGO plug-in and listed according to their enrichment *p*-value (*p* < 0.05). (**e**–**h**) Results of candidate gene expression analysis performed by RT-qPCR assay. Expression profiles of key genes belonging to cluster 3 (**e**-*VvOPR3*, VIT_11s0016g01230; **f**-*VvNCED*, VIT_19s0093g00550), and cluster 4 (**g**-*VvCHIT*, VIT_04s0008g00140, **h**-*VvMLO6*, VIT_08s0040g02170) analyzed in leaf samples taken from ‘Nebbiolo’ and ‘Moscato’ plants artificially inoculated with *Erysiphe necator* and then untreated (CTR) or treated with acibenzolar-S-methyl (AcS-Mt), potassium phosphonate (K-Pho), and laminarin (Lam). Ubiquitin (*VvUBI*) and Actin (*VvACT1*) genes were both used as endogenous controls for the normalization of transcript levels. Significance of genotype, treatment, and genotype × treatment (G × T) interaction was assessed by Tukey’s HSD test for all *p* values and the corresponding results are given above each graph in the figure panel; *, **, **** indicate *p* ≤ 0.05, *p* ≤ 0.01, and *p* ≤ 0.0001, respectively; n.s. = not significant. Lower case letters above bars are reported when the G × T interaction and/or Genotype (G) main effects are statistically significant as attested by Tukey’s HSD or Student’s *t*-test, respectively. Error bars represent SE. Three independent biological replicates with three technical replicates each were used for the analysis.

A further elaboration of HCL data attested that the 6082 DEGs were resolved to four major clusters: cluster 1 was characterized by 1777 genes that were significantly down-regulated by AcS-Mt (Figure 2a, Table S3); cluster 2 included 1544 genes that were instead positively affected by AcS-Mt application (Figure 2b, Table S3); cluster 3 grouped 1698 genes exclusively up-regulated in ‘Moscato’ leaf (Figure 3a, Table S3), whereas cluster 4 included the remaining 1063 genes that were exclusively up-regulated in ‘Nebbiolo’ samples (Figure 3b, Table S3). This DEG clusterization suggested that the genotype (‘Moscato’ and ‘Nebbiolo’) and AcS-Mt treatment were the variables that, in our experimental conditions, most significantly impacted on the leaf transcriptome modulation.

### 2.1. Treatment with AcS-Mt Induces an Extensive Transcriptome Reprogramming in PM-Infected Leaves

Clusters 1 and 2 enclosed transcripts significantly modulated in response to AcS-Mt, regardless of the cultivar (Figure 2a,b). The Gene Ontology (GO) enrichment analysis conducted on transcripts belonging to cluster 1 indicated that carbohydrate metabolism, photosynthesis, energy metabolism, secondary metabolism, and biosynthetic process were the overrepresented functional categories (Figure 2c). Consistently, a closer look to AcS-Mt downregulated genes revealed a high number of transcripts encoding enzymes involved in the Calvin cycle (e.g., glyceraldehyde-3-phosphate dehydrogenase, fructose-bisphosphate aldolase, phosphoglycerate kinase, ribose-5-phosphate isomerase, and ribulose-phosphate 3-epimerase), in the photosynthetic process (e.g., chlorophyllase, light-harvesting complex (LHCII), and photosystem I and II subunit proteins) and in starch and sucrose metabolism (e.g., starch synthase, beta-amylase, and sucrose synthase) (Table S3). Additionally, the application of AcS-Mt to PM-infected plants decreased the expression of many genes involved in phenylpropanoid biosynthetic processes (e.g., flavonoid 3′ and 3’,5’-hydroxylase, flavonoid 3 monooxygenase, and stilbene synthase) (Table S3). These expression trends were confirmed by the outcomes of Real-Time PCR assays performed on a group of selected genes (Figure S1). Transcripts encoding chlorophyllase (*VvCHL*) and starch synthase (*VvSTA*) underwent a statistically significant downregulation in AcS-Mt-treated leaves (Figure 2e,f), sharing transcriptional profiles similar to other photosynthesis-related genes, like transcripts for photosystem II subunits (*VvPSBx*, Figure S2a).

Protein metabolism and modification, transport, and cell communication were the enriched functional categories within genes grouped in cluster 2, which were upregulated in response to AcS-Mt (Figure 2d). Particularly, several protein kinase- and serine/threonine kinase-encoding transcripts were overexpressed in these samples, together with genes encoding heat shock proteins and ubiquitin-conjugating enzymes (Table S3). Interestingly, proteins involved in stress responses were enhanced in AcS-Mt-treated samples, as highlighted by the activation of genes encoding disease resistance proteins, glutathione S-transferases, and R proteins, in addition to a number of stress-dependent transcription factors, like WRKY, zinc finger, and NAC domain-containing proteins (Table S3). Finally, independently of the cultivar, many transporter-encoding genes were transcriptionally activated in PM-infected leaves following treatment with AcS-Mt. These included ABC and ABCG transporters, such as the *Vitis* homolog to the *Arabidopsis* abscisic acid (ABA) transporter ABCG40 [42], amino acid permeases, and different genes involved in the transport of sugars, such as the grape hexose transporters *VvHT*2 and *VvHT5* [43], and glucose-6-phosphate/phosphate translocators (Table S3). Accordingly, results achieved through candidate gene expression analysis attested that invertase genes, such as the cell wall invertase *VvCW-INV* (Figure 2g) and the acidic vacuolar invertase *VvGIN2* (Figure S2c), were strongly induced upon AcS-Mt treatment, as well as sugar transport transcripts (*VvHT5*, Figure 2h and *VvHT2*, Figure S2d). These findings are in line with previously published information evidencing a role for these genes in molecular signaling cascades enhancing grapevine defense to different pathogens [44,45,46], including biotrophic fungi [43].

### 2.2. Dissection of Molecular and Metabolic Changes Triggered by the Genotype Effect

Analysis of genes belonging to clusters 3 and 4 underlined the presence of cultivar-specific molecular responses that were independent of elicitor application (Figure 3a,b). The GO enrichment analysis applied on cluster 3 DEGs yielded only one functional class, i.e., secondary metabolic processes (Figure 3c). Within this group, most genes encoded enzymes involved in terpenoid and phenylpropanoid metabolism, such as cinnamyl alcohol dehydrogenases, flavonol synthases, isoflavone methyltransferases, CYP P450 monooxygenases, and several laccases (Table S3).

The list of genes exclusively activated in PM-infected ‘Moscato’ samples also included many transcripts belonging to hormone biosynthesis and hormone-related signaling cascades. Accordingly, genes linked to ABA (e.g., 9-cis-epoxycarotenoid dioxygenase, ABA 8’-hydroxylase, and ABA-glucosyl-transferase), Jasmonate (JA) (e.g., 12-oxophytodienoate reductase, jasmonate ZIM domain proteins, jasmonate O-methyltransferase, and lipoxygenases), ethylene (e.g., 1-aminocyclopropane-1-carboxylate oxidase and ethylene-responsive protein), and auxin (e.g., expansin and auxin-responsive factors) signaling were all overexpressed (Table S3).

Real-Time PCR analysis of JA (*VvOPR3*; Figure 3f) and ABA (*VvNCED*; Figure 3g) biosynthetic genes, as well as of stilbene synthase (*VvSTS48*) and laccase encoding genes (*VvLAC14*; Figure S2e), evidenced the prevalent induction of such secondary metabolic processes in PM-infected ‘Moscato’ plants. Also deserving of attention in ‘Moscato’ is the statistically significant upregulation of the *PR1* gene (Figure S2f), encoding a pathogenesis-related protein associated with elicitor-dependent priming of defense response [39]. However, in our experimental conditions, we found that this gene was most predominantly modulated by genotype instead of treatment, as emerged by comparing the two cultivars even in absence of elicitor application (CTR) (Figure S2f).

The analysis of genes mainly activated in ‘Nebbiolo’ leaves and included in cluster 4 highlighted a completely different reprogramming of defense responses than what was observed in ‘Moscato’ (Figure 3b). In this cluster, regardless of the antifungal compound used, the significantly over-represented gene categories were protein metabolism, cell death, cell communication, and response to biotic stimulus (Figure 3d). The majority of genes assigned to these functional classes encoded proteins linked to stress perception (e.g., cell wall-associated receptor kinases and kinases, S receptor serine/threonine kinase and protein kinases) and defense cellular processes, typically activated in the presence of pathogen attack, including chitinases, xyloglucan endotransglucosylases, disease resistance proteins, peroxidases, glutathione S transferases, thaumatins, accelerated cell death, and pathogenesis-related proteins (Table S3). This trend clearly emerged by analyzing transcriptional levels of a chitinase gene that was up to four times more expressed in PM-infected ‘Nebbiolo’ than ‘Moscato’ samples (*VvCHIT*, Figure 3g).

Although genotype-dependent transcriptional reprogramming plays a central role in channeling distinct defense pathways in the analyzed cultivars, another important outcome is that the genotype × treatment (G × T) combination differently affects the nature of transcriptional and metabolic signals raised against the pathogen. This aspect is particularly evident in looking at transcriptional profiles of the nitrite/nitrate transporter NPF3 (*VvNPF3*), homolog to the *Arabidopsis* NPF3 [47], which is typically activated in grapevine under *Erysiphe necator* infection [48]. *NPF3* was significantly more expressed in ‘Nebbiolo’ than ‘Moscato’ but exclusively after the treatment with Lam (Figure S2b). Another example of the G × T effect is represented by differences in the regulation of some hormone-related genes, such as *VvNCED*. In ‘Nebbiolo’, transcripts of this gene were overexpressed in AcS-Mt and K-Pho-treated samples but downregulated following Lam treatment as compared to CTR. By contrast, in ‘Moscato’ the same gene was always up-regulated in treated than in untreated leaves, and particularly in the case of AcS-Mt (Figure 3f). Mildew Locus O protein genes (*MLO6*), directly associated with grapevine susceptibility to PM disease [48], were much more expressed in ‘Nebbiolo’ than in ‘Moscato’, irrespective of treatment application. Nevertheless, comparing the two cultivars, the same resistance inducer differently affects the transcription of these genes: in comparison with CTR leaves, K-Pho was the compound that most effectively reduced *MLO6* levels in ‘Nebbiolo’, while in ‘Moscato’ a similar effect was noticed for Lam, although at a lesser extent (Figure 3h).

### 2.3. Defense Secondary Metabolites Are Differentially Accumulated in ‘Moscato’ and ‘Nebbiolo’

Changes associated with transcriptional reprogramming of stress response were then integrated with the analysis of secondary metabolic compounds and hormones acting as defense mediators. Based on transcriptomic data elaboration, attention was given to endogenous concentrations of epicuticular waxes, stilbenoids, including piceid forms, resveratrol and its derivatives, and stress-associated phytohormones, such as ABA, JA, and SA.

**Figure 4:**
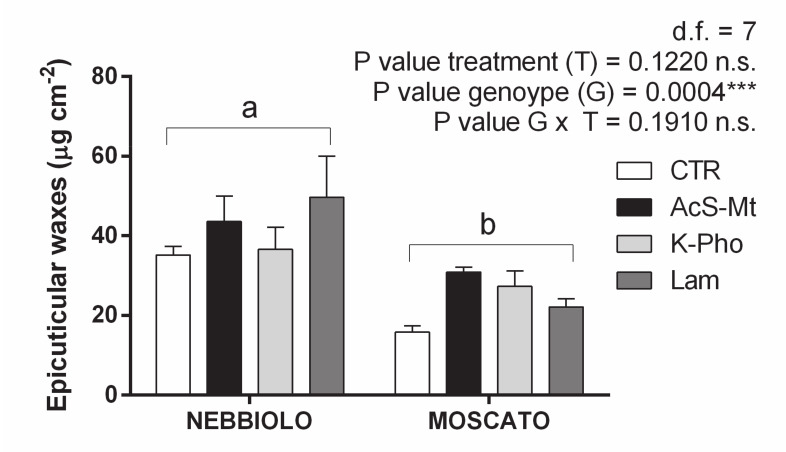
Differences in epicuticular wax deposition. Content of epicuticular waxes (µg cm^−2^) present on leaves of ‘Nebbiolo’ and ‘Moscato’ plants artificially inoculated with *Erysiphe necator* and then untreated (CTR) or treated with acibenzolar-S-methyl (AcS-Mt), potassium phosphonate (K-Pho), and laminarin (Lam). Significance of genotype, treatment, and genotype × treatment (G × T) interaction was assessed by Tukey’s HSD test for all *p* values and the corresponding results are given above each graph in the figure panel; *** indicates *p* ≤ 0.001 and n.s. = not significant. Lower case letters above bars denote the significance of the Genotype (G) main effects as attested by the Student’s *t*-test. Error bars represent SE (*n = 3*).

In ‘Nebbiolo’, wax amounts were significantly higher than ‘Moscato’, regardless of treatment application, suggesting that deposition of epicuticular waxes was mainly influenced by the cultivar (Figure 4). Accordingly, the expression profiles of many genes involved in epicuticular wax accumulation were predominantly modulated by the genotype component. For instance, the majority of transcripts linked to lipid transport (e.g., ABC and ABCG transporters, and lipid transfer proteins) and synthesis of epicuticular wax precursors (e.g., CER1, wax synthase, 3-ketoacyl-CoA reductase, Acyl-CoA oxidase, and 3-ketoacyl-CoA thiolase), such as long-chain fatty acids and primary alcohols, fell within the group of ‘Nebbiolo’ induced genes ascribed to cluster 4 (Table S3).

**Figure 5:**
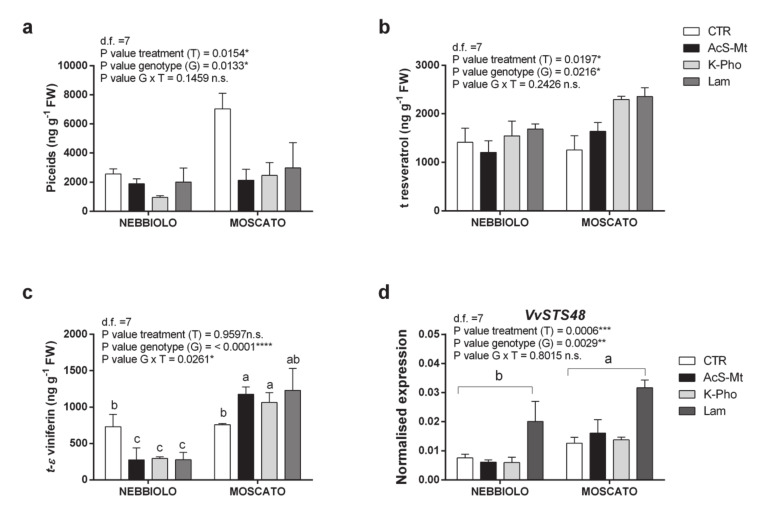
Accumulation of antimicrobial compounds. Accumulation patterns of (**a**) piceid forms, (**b**) t-resveratrol, and (**c**) t-*ε*-viniferin in leaf samples taken from ‘Nebbiolo’ and ‘Moscato’ plants artificially inoculated with *Erysiphe necator* and then untreated (CTR) or treated with acibenzolar-S-methyl (AcS-Mt), potassium phosphonate (K-Pho), and laminarin (Lam). RT-qPCR expression profiles of the stilbene synthase-encoding gene *VvSTS48* (VIT_16s0100g01200) are also displayed for the same samples (**d**). Significance of genotype, treatment, and genotype × treatment (G × T) interaction was assessed by Tukey’s HSD test for *p* ≤ 0.05 (*), *p* ≤ 0.01 (**), *p* ≤ 0.001 (***), and *p* ≤ 0.0001 (****) and the corresponding results are given above each graph in the figure panel; n.s. = not significant. Lower case letters above bars are reported when the G × T interaction and/or genotype (G) main effects are statistically significant as attested by Tukey’s HSD or Student’s *t*-test, respectively. Error bars represent SE (*n = 3*).

Intriguingly, the accumulation of antimicrobial compounds, such as phytoalexins, was reversed in the two cultivars. In the absence of treatment, PM-infection significantly increased piceid forms in ‘Moscato’ but not in ‘Nebbiolo’ (Figure 5a). Additionally, in ‘Moscato’, the application of K-Pho and Lam significantly stimulated the synthesis of *t*-resveratrol (Figure 5b). Interestingly, the genotype effect and the importance of the G × T interaction rather emerged from the analysis of *t*-*ε*-viniferin, a resveratrol derivative whose role as an antimicrobial compound, besides against other fungi [49], has been experimentally proved in grapevine exposed to *E. necator* infection [50]. Viniferin concentrations were significantly higher in ‘Moscato’ than in ‘Nebbiolo’ in the presence of all tested elicitors (Figure 5c). The ‘Moscato’-driven increase in stilbenoid amounts found a molecular correspondence in the expression profiles of stilbene synthase genes, as *VvSTS48* was significantly more expressed in ‘Moscato’ than in ‘Nebbiolo’ (Figure 5d). However, a treatment effect was also highlighted for this gene, as in both cultivars, *VvSTS48* transcriptional levels were two-fold higher in Lam-treated than in other samples, including CTR (Figure 5d).

**Figure 6:**
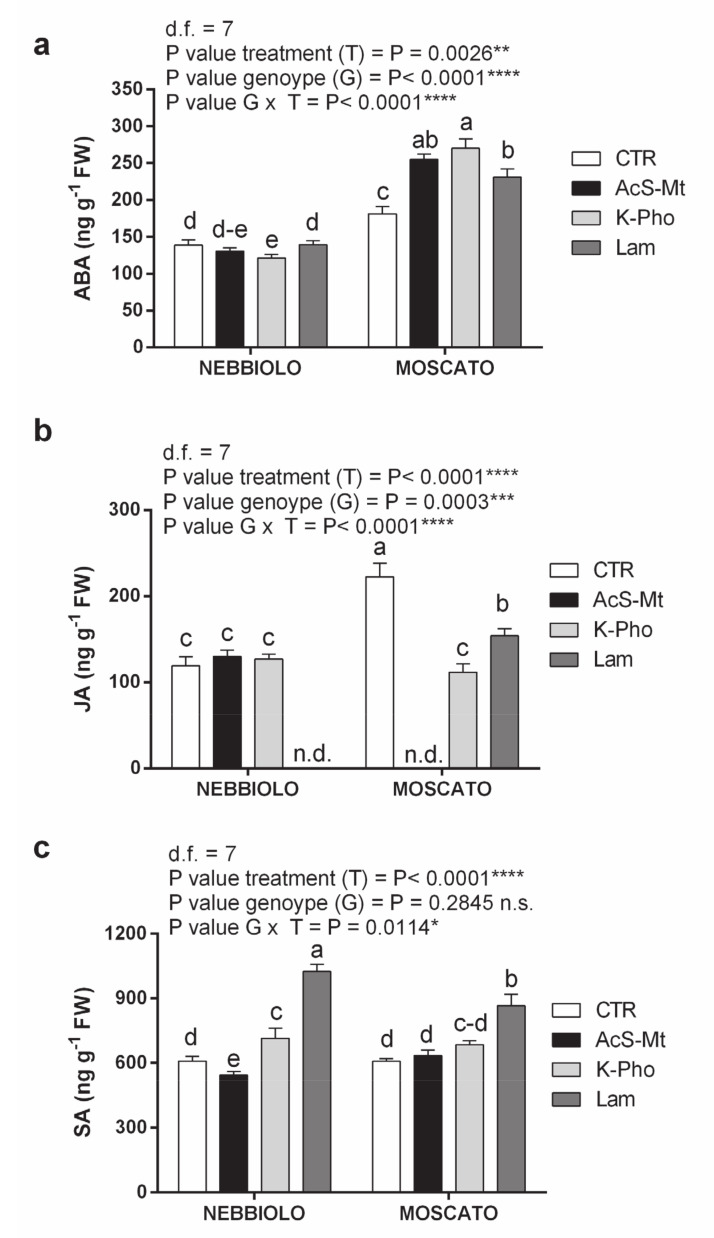
Content of stress-associated phytohormones. Changes in the concentration levels of endogenous (**a**) abscisic acid (ABA), (**b**) jasmonic acid (JA), and (**c**) salicylic acid (SA) in leaf samples taken from ‘Nebbiolo’ and ‘Moscato’ plants artificially inoculated with *Erysiphe necator* and then untreated (CTR) or treated with acibenzolar-S-methyl (AcS-Mt), potassium phosphonate (K-Pho), and laminarin (Lam); n.d. not detected. Significance of genotype, treatment, and genotype × treatment (G × T) interaction was assessed by Tukey’s HSD test for *p* ≤ 0.05 (*), *p* ≤ 0.01 (**), *p* ≤ 0.001 (***), and *p* ≤ 0.0001 (****) and the corresponding results are given above each graph in the figure panel; n.s. = not significant. Lower case letters above bars are reported when the G × T interaction and/or genotype (G) main effects are statistically significant as attested by Tukey’s HSD or Student’s *t*-test, respectively. Error bars represent SE (*n = 3*).

Effects due to the considered variables were particularly evident when concentrations of ABA, JA, and SA were analyzed in the two cultivars (Figure 6). For instance, genotype was the component that mostly affected ABA contents, which were significantly higher in ‘Moscato’ than in ‘Nebbiolo’, even in absence of treatment (Figure 6a). These data supported at the metabolic level the strong activation of the ABA biosynthetic gene *NCED* in ‘Moscato’ leaves (Figure 3). While in ‘Moscato’ ABA concentrations were positively affected by all applied treatments, and particularly by AcS-Mt and K-Pho, in ‘Nebbiolo’ the same resistance elicitors did not affect or even reduce, endogenous ABA levels with respect to CTR condition (Figure 6a), thus highlighting the influence of the G × T interaction. Samples collected from CTR plants of ‘Moscato’ were also particularly enriched in JA, with values more than two-fold higher than in ‘Nebbiolo’ CTR leaves (Figure 6b). All treatments did significantly reduce JA concentrations in ‘Moscato’, while in ‘Nebbiolo’ a similar result was only observed in the case of Lam application. Moreover, with the exception of K-Pho, the resistance inducers had the opposite effect on the JA levels of the two cultivars: JA was not detectable in AcS-Mt-treated leaves of ‘Moscato’, the same happened to ‘Nebbiolo’ but under Lam application (Figure 6b), underlining the strong effect due to the G × T interplay. Finally, a strong accumulation of SA was noticed in Lam-treated samples of both cultivars (Figure 6c).

## 3. Discussion

### 3.1. Long-Term Defense Responses Are Induced Following Application of Resistance Elicitors

The molecular scenario emerging from the elaboration of RNAseq data indicated that AcS-Mt is the compound that causes the most intense transcriptome perturbation in PM-infected leaves, regardless of genotype-associated variability. The other tested products, K-Pho and Lam, modify the plant metabolism less profoundly than AcS-Mt, as attested by the very low number of significantly differentially expressed genes resulting by comparing K-Pho- and Lam-treated leaf transcriptomes with CTR samples, and by clustering analysis. We previously showed that K-Pho and Lam are both effective in controlling PM in ‘Moscato’ and ‘Nebbiolo’ vines [40]. Therefore, it cannot be excluded that these compounds could activate some early leaf defense responses able to limit the pathogen proliferation in the hours immediately after the treatment application. For instance, the fact that, regardless of genotype, SA concentrations were much higher in K-Pho- and particularly in Lam-treated leaves than in CTR and AcS-Mt samples could represent a reminiscence of early-response metabolic signals. Laminarin was indeed shown to induce early defense genes involved in SA-signal transduction and secondary metabolism (e.g., MAP kinases, PR proteins, PAL, and LOX) in *V. vinifera* cv Gamay suspension cells, even within a few hours from the treatment [51]. However, such aspect was not investigated further in our experiments since we were interested in exploring long-term, rather than early, molecular responses, in order to uncover a) functional associations with priming events triggered during the previous infection, and b) treatment-specific responses.

It has been reported that synthetic resistance inducers analogous to natural hormones, such as AcS-Mt, boost the plant resistance system against pathogen attack by triggering SAR [30]. This consequently leads to transcriptional activation of pathogenesis-related proteins (PR proteins), including PR-1, β 1,3 glucanases, and chitinases, as well as cell wall modifying enzymes, within the first two to three days after pathogen inoculation [39]. Interestingly, AcS-dependent transcriptional modifications were not associated here with significant increases of SAR-associated gene expression or SA content, which in both cultivars were not significantly different than CTR and much lower than those measured in Lam. The main hallmark of AcS-Mt transcriptome is rather a profound reprogramming of primary metabolic genes involved in photosynthesis and carbohydrate accumulation and partitioning. This may suggest a transition in the growth–defense trade-off established through a reallocation of the endogenous resources in favor of the activation of the immune response [52]. Accordingly, genes involved in photosynthesis and carbohydrate and energy metabolism were globally inhibited in AcS-Mt treated leaves with respect to other conditions, whereas processes tied to sugar transport and cell communication were induced. Strong inhibition of the photosynthetic processes is a basic response in compatible plant-biotrophic microorganism interactions, and it is established in coordination with the accumulation of soluble sugars in the leaves, supported by the enhanced expression of genes encoding sugar mobilizing enzymes [53]. Changes to photosynthesis and carbohydrate metabolism in different tissues also represent a basic physiological response of grapevine, not only in presence of PM attack [35,43,54] but also under other plant diseases [34,44,45]. The enhanced activity of grape invertase genes coupled with the increased accumulation of soluble sugars in infected leaves most likely causes a shift in the sink-source balance that progressively slows down the Calvin cycle, with consequent serious reduction of the plant assimilation rates [44,45,46,55]. Although carbohydrate metabolism is globally down-regulated by AcS-Mt treatment in our samples, other specific categories of carbohydrate metabolic genes, mostly involved in sugar-primed signaling processes, were highly activated. Particularly, expression of genes encoding starch degrading enzymes (e.g., b-amylases, AGPases, and starch synthases) was overall inhibited in association with that of Rubisco, PSI, and PSII chlorophyll-binding proteins and other genes related to photosynthesis. Conversely, cell wall invertases, apoplastic invertases, and alpha-amylases fell within the AcS-Mt induced genes together with transcripts encoding sugar transporters of the HT family. Transport of sugars is significantly altered by biotic stress, as a defense strategy against fungal pathogens that typically need access to the host sucrose reserves to complete their development [56]. Hence, transcriptional regulation of sugar metabolism may help the plant to support the cell osmotic and metabolic functions altered by the disease. However, soluble sugars are also involved in cell communication pathways that could lead to the activation of a defense response via interaction with hormonal pathways, such as those mediated by SA [57]. Being AcS-Mt a synthetic functional analog of SA, it is likely that the transcriptional activation of hexose transporter and invertase genes could facilitate the establishment of a sugar signaling network leading to increased transcription of disease resistance genes (“sweet priming”). In support of this hypothesis, Hayes and coll. [43] showed that, in ‘Cabernet Sauvignon’ plants exposed to PM infection, mobilized sugars could transcriptionally regulate the expression of defense-related genes in sink organs and that of photosynthesis-related genes in source organs. Moreover, among the analyzed stress-inducible HT transporters, these authors reported that *HT5*, as well as *cw-INV*, is transcriptionally activated by ABA. Accordingly, in our experiment, *NCED* expression was induced by AcS-Mt in both cultivars, resembling the transcriptional profiles of *HT5* and *cw-INV* in the same samples. However, at the metabolic level, the AcS-Mt enhancing effect on ABA synthesis was visible only in ‘Moscato’ leaves, consistently with the predominant overexpression of genes related to the ABA biosynthetic pathway observed in this cultivar.

The above-discussed framework of molecular and metabolic responses thus supports the existence of long-lasting AcS-Mt biological effects that, by stimulating the coordinated activity of ABA and sugar signaling pathways, could facilitate the maintenance of the plant defense status previously primed during the infection.

### 3.2. Genotype-Dependent Metabolic Signals Are Crucial to Orient the Plant Defense Machinery

It is known that metabolic responses to *Erysiphe necator* can vary greatly among *Vitis* species [9], but information on intraspecies variability-mediated phenomena involved in the regulation of either susceptibility or resistance to this pathogen is still limited. Conversely, this subject was widely explored in relation to grape downy mildew [58,59].

Disease index data from our previous survey highlighted significant differences between the white-grape Moscato and the red-grape Nebbiolo varieties in terms of susceptibility to PM, even in absence of elicitor treatments [40]. To test the hypothesis that intraspecies variability could stimulate specific defense strategies, we searched for genotype-dependent molecular and metabolic changes by comparing infected untreated and treated leaf tissues from these varieties, paying attention to the significant effects due to the G × T interaction.

The overall greater tolerance of ‘Moscato’ observed after treatment with the resistance elicitors could be associated with a basal constitutive activation of genes linked to hormone and secondary metabolism, as highlighted by increased transcriptional rates of JA and ABA metabolic genes, laccases, and *STS48*—the only one among *STS* genes to be overexpressed in ‘Moscato’ rather than ‘Nebbiolo’, and potentially leading to the increased production of specific stilbenoids, such as viniferin. Indeed, unlike piceid and resveratrol, contents of viniferin are significantly affected by the G × T interaction, showing an opposite response between the cultivars. This finding is particularly interesting in the perspective of in-depth investigations on genotype-primed defense signals associated with resistance inducers, consistently with previous results reporting AcS-Mt- and Lam-driven accumulation of viniferin in response to both *E. necator* and *P. viticola* [39,51]. Interestingly, viniferin is the phytoalexin that probably contributes the most to elicit the plant resistance system against diseases [50,60], and, on a wider perspective, an increased constitutive viniferin production may be at the basis of grapevine immunity responses [61]. It is thus feasible that, in ‘Moscato’, high constitutive basal levels of antimicrobial metabolites contribute to successfully prime long-term resistance mechanisms in agreement with the very low transcriptional rates of *MLO* genes observed in this cultivar.

It deserves attention the fact that, although ‘Nebbiolo’ accumulates significantly fewer stilbenoid amounts than ‘Moscato’, even in absence of treatment *STS* genes (with the exception of *STS48*) are highly transcribed in this cultivars leaves, suggesting a delay regulatory activation of this metabolic route compared with ‘Moscato’. This feature associated with high transcriptional rates of many PR protein genes, including chitinases, and by the significantly high accumulation of leaf epicuticular waxes, underlines the typical defense strategy triggered in grapevine to limit *E. necator* proliferation [62,63]. Notably, the observed pattern of transcriptional and metabolic changes is consistent with pathological data showing a higher disease severity in untreated ‘Nebbiolo’ than in untreated ‘Moscato’ leaves, and with ITS2 sequencing results showing a higher abundance of *Erysiphe* genus sequences in the same samples regardless of treatment [40]. The fact that in ‘Nebbiolo’ stress-responsive genes (e.g., MLO, NBS-LRR proteins, chitinases, and STS) were so highly expressed in comparison with ‘Moscato’ attests that the basal defense status of these plants against the pathogen is still active, resembling short-term transcriptional changes reported for other grape cultivars and *Vitis* species in the presence of PM [33,36]. The upregulation of grape *MLO6* transcripts is considered a molecular marker of susceptibility to PM, and the knockdown of this gene in ‘Brachetto’ plants caused a significant reduction of PM susceptibility in the transgenic lines [48]. Accordingly, the overexpression of *MLO6* transcripts in PM-infected ‘Nebbiolo’ leaves could be associated with an intrinsic greater sensitivity of this variety to the pathogen, which is still evident in the transcriptome of treated samples.

The abovementioned molecular signals are very weak in ‘Moscato’, in which we rather assist to a strong reprogramming of secondary metabolic pathways accompanied by activation of ABA-mediated signaling networks. It was furthermore documented that ABA metabolic and signaling pathways can vary depending on the plant-pathogen pathosystem and on timing [64], resulting in either induction (early response) or limitation (delay response) of plant defense signals by antagonizing the effect of other hormones, like SA and ethylene [65]. This may explain, at least partly, the ABA-related transcriptional and metabolic differences observed between the two varieties as well as the less variation in SA contents observed in ‘Moscato’ among the tested conditions.

The transcriptional reprogramming events occurring in ‘Moscato’ leaves are thus translated at the biochemical level into the accumulation of antimicrobial substances, in particular of viniferin, the toxic derivative of trans-resveratrol [49], and stress-related hormones (ABA), ultimately supporting the reduced disease severity symptoms noticed in this cultivar [40]. This most likely suggests that the long-term reprogramming of secondary metabolism due to the genotype intrinsic features in combination with specific treatment (e.g., Lam-mediated over-accumulation of viniferin) may serve to stimulate innate immunity response in ‘Moscato’. By contrast, the basal defense status experienced by ‘Nebbiolo’ points to a delay in early metabolic responses typically established by PM susceptible cultivars [37], and likely underlying the higher sensitivity of this cultivar to the disease [40]. However, it cannot be excluded here that on a longer time span the defense-oriented reprogramming of metabolism observed in ‘Nebbiolo’ could prime the plant immunity system to stimulate similar signaling pathways as observed in ‘Moscato’.

Collectively, these results evidence that the two cultivars have built up different reactions to the pathogen, often enhanced by the effect of a specific resistance elicitor, and depending on either reinforcement of early defense mechanisms (‘Nebbiolo’) or activation of molecular routes involving hormone and secondary metabolites able to prime endogenous immune responses (‘Moscato’). It thus emerges that the key to advancing effective protection strategies against grape pathogens is a thorough understanding of the biological dynamics characterizing the interaction between elicitor- and genotype-based molecular and metabolic responses.

Although more efforts are undoubtedly necessary to achieve a comprehensive view of the intraspecies-variability effects featuring the plastic response of diseased plants to resistance elicitors, these data provide a first outlook on the molecular bases controlling the tripartite interaction among genotype, pathogen, and resistance inducers in grapevine. Future experiments on this subject-for instance, by testing different elicitor-cultivar combinations either in presence of the same or other fungal pathogens-would help to provide additional deepening on the biological effects of resistance elicitors associated with plant disease control. Notably, outcomes from such studies would also facilitate the development of more sustainable pest management programs in viticulture, as they would provide useful information for orienting the choice of resistance inducers in the field, based on the specific genotype-pathogen combination.

## 4. Materials and Methods

### 4.1. Plant Material and Experimental Set Up

The trials were carried out from August to September in 2017 following the experimental set up previously described in [40]. Briefly, one-year-old grapevines (*Vitis vinifera* L.) of ‘Moscato’ CN 4 clone (white grape variety) and ‘Nebbiolo’ CVT CN 142 clone (red grape variety) grafted onto Kober 5BB were grown in pots and maintained in an open-air environment system 100 m from an experimental vineyard, located at the Centre AGROINNOVA of the University of Torino (Grugliasco, Italy), in which PM infections naturally occurred. The adopted experimental set up was conceived to resemble as much as possible the natural vineyard conditions and allowed to study the treatment effect(s) on target plants by reducing issues other than those here studied, typical of a completely open field experimental design (e.g., abiotic factors as well as attacks by insect and other pathogens) and potentially altering the analyzed plant responses.

Plants were artificially inoculated with a suspension of 1 × 10^5^ conidia/mL of *Erysiphe necator*, and in addition to an infected untreated control (CTR), three commercial formulations were applied according to manufacturer’s instructions: acibenzolar-S-methyl (Bion, Syngenta Crop Protection) (AcS-Mt), potassium phosphonate (Century, BASF Agro) (K-Pho), and laminarin (Vacciplant, Arysta Lifescience) (Lam) [40]. Two consecutive experiments were carried out working on a total of 36 ‘Moscato’ and 36 ‘Nebbiolo’ vines. The first trial was conducted using 20 plants per cultivar (5 plants × 4 treatments, the latter corresponding to the three commercial products and the untreated control), while the second one was carried out on 16 plants per cultivar (4 plants × 4 treatments). Each plant was used for the trials 60 days after transplanting the cuttings. Product application and artificial inoculation with *Erysiphe necator* were performed as described in [40] (Table S4). The plants used as CTR were inoculated with *Erysiphe necator* without applying products for controlling the pathogen before and after the inoculation (Table S4). Data on disease severity (% of leaf surface affected) and incidence (% of affected leaves) were collected after the last treatment, at the onset of symptoms, as reported in [40]. For molecular and metabolic assays, three independent biological replicates were obtained for each treatment by pooling six asymptomatic leaves randomly selected from three plants (2 leaves × 3 plants × 3 biological replicates: each set of three plants represented an independent biological replicate).

As we wanted to focus our survey on the study of genotype- or treatment-dependent long-term molecular priming responses, the sampling was conducted three days after the last treatment, which corresponded to 17 days after pathogen inoculation (Table S4). Once collected, the leaf samples were immediately frozen in liquid nitrogen and stored at −80 °C until use.

### 4.2. RNA Extraction, Sequencing, and Bioinformatic Elaboration of Data

Total RNA was extracted starting from 100 mg of leaf material using the Spectrum^TM^ Plant Total RNA kit (Sigma-Aldrich Co., MI, USA), following the manufacturer’s instruction. Total RNA yield and purity were determined using a NanoDrop 2000 spectrophotometer (Thermo Fisher Scientific, Waltham, MA, USA) and integrity was checked at the 2100 Bioanalyzer (Agilent Technologies, Waldbronn, Germany). Only samples showing an RIN (RNA Integrity Number) value higher than 8 were submitted to sequencing and quantitative expression analyses. RNA libraries were obtained using the TruSeq RNA Library V2 kit and sequenced onto a NovaSeq Illumina apparatus by an external service (Macrogen, Inc., South Korea) following a paired-end approach. Reads generated by sequencing the 24 cDNA libraries (three biological replicates for each condition) were trimmed by Trimmomatic v0.38 and quality checked by FastQC (v0.9, Bioinformatics Group, The Babraham Institute, Cambridge, CB22 AT, UK). The Bowtie software (v1.1.2, The Johns Hopkins University, Baltimore, MD, USA) with one mismatch allowed in the alignment was used to map the reads onto the V1 transcriptome derived from the *V. vinifera* assembly 12X [66]. For the identification of differentially expressed genes (DEGs), the DESeq2 package (v.1.14.1, Bioconductor, Roswell Park Comprehensive Cancer Center, Buffalo, NY, USA) was run on a 112 core and 512 GB RAM local machine, using Ubuntu server 16.04.6 LTS. DEG selection was based on an adjusted *p*-value ≤ 0.01. Transcripts were annotated using the V1 version of the 12X draft annotation of the grapevine genome [67] and grouped into functional gene classes according to the GO biological process classification and to the VitisNet GO annotations [68]. The log2 transformed FPKM values deriving from RNAseq data elaboration were then used as input of hierarchical clustering (HCL) analysis, which was conducted by applying the Pearson’s correlation distance and the MeV software (v4.9, Dana-Farber Cancer Institute, Boston, MA, USA). GO enrichment analysis was carried out using the BiNGO 3.0 plug-in tool in Cytoscape (v3.2, U.S. National Institute of General Medical Sciences (NIGMS), Bethesda, MD, USA), as described by [69], and over-represented Plant GO slim categories were identified using a hypergeometric test with a significance threshold of 0.05.

### 4.3. Real-Time PCR Analysis

Expression changes of key candidate transcripts were quantitatively determined by Real-Time PCR assay (RT-qPCR) on the same RNA samples used for RNAseq analysis. Besides being addressed to validate RNAseq results, this analysis allowed us to understand more in-depth the impact of the studied factors (treatment, genotype, and their interaction) on the regulation of specific genes.

For each biological replicate, RNA samples were treated with DNase I (Invitrogen, Thermo Fisher Scientific Waltham, MA, USA), and first-strand cDNA was synthesized starting from 500 ng of total RNA using the High Capacity cDNA Reverse Transcription kit (Applied Biosystems, Thermo Fisher Scientific Waltham, MA, USA) following the manufacturer’s instructions. Gene-specific primers (Table S5) were designed using the Primer Express R software (v3.0, Applied Biosystems, Thermo Fisher Scientific Waltham, MA, USA). Reactions were carried out in a CFX Connect Real-Time PCR system (Bio-Rad Laboratories, Hercules, CA, USA), using SYBR Green (iQTM SYBR Green Supermix; Bio-Rad Laboratories, Hercules, CA, USA) for quantifying the amplification results. Thermal cycling conditions were as follows: an initial denaturation phase at 95 °C for 2 mins, followed by 40 cycles at 95 °C for 15 s and 60 °C for 30 s. At the end of each RT-qPCR run, the specificity of primer annealing was inspected through the dissociation kinetics curve. Three technical replicates were run for each of the three independent biological replicates, and the geometric mean of the expression ratios of two housekeeping, ubiquitin (*VvUBI*) and actin1 (*VvACT1*), was used to normalize transcript expression levels.

### 4.4. Measurement of Epicuticular Wax Content

Epicuticular waxes were extracted according to previously published methods [63,70] with slight modifications. Briefly, fresh whole leaves were dipped into chloroform (1.5 mL/2.5 cm^2^) and gently shaken for 1 min at room temperature. The solvent was then dropped into a glass Petri dish and allowed to completely evaporate under the hood for 24 h before weighting extracts. The net weight of chloroform extractable material was calculated by subtracting the empty Petri weight, measured before the extraction, from the Petri weight measured after extraction. For each tested condition, three samples, each constituted by three leaves, were collected and used for the analysis. Before the extraction, the total leaf surface area (LA) was determined for each leaf sample using the ImageJ software (v2020, National Institutes of Health and the Laboratory for Optical and Computational Instrumentation, LOCI, University of Wisconsin-Madison, USA), thus the wax amount was referred to LA and expressed as µg per cm^2^.

### 4.5. Analysis of Secondary Metabolites

Contents of piceids, trans-resveratrol, and trans-*ε*-viniferin were quantified on three biological replicates per condition starting from 500 mg of powdered leaves and according to the protocol described in [71]. Briefly, homogenized samples were transferred in a 2 mL centrifuge tube with 1 mL of methanol:water (1:1 *v/v*) acidified with 0.1% of formic acid in an ultrasonic bath for 1 h. Samples were centrifuged at 15,000 rpm and 4 °C for 2 min, and the supernatant was analyzed by HPLC-DAD technique.

The original standard of polydatin (purity ≥95%), resveratrol (purity ≥99%), and viniferin (purity ≥95%), purchased from Sigma-Aldrich, were used for the identification of the studied metabolites by comparing retention times and UV spectra. The quantification was made by the external calibration method. The HPLC apparatus was an Agilent 1220 Infinity LC system model G4290B (Agilent^®^, Waldbronn, Germany), equipped with a gradient pump, autosampler, and column oven set at 30 °C. A 170 Diode Array Detector (Gilson, Middleton, The USA) set at 265 nm (for ABA) and 280 nm (for stilbenes) was employed. A XTerra RP18 analytical column (150 × 4.6 mm i.d., 5 µm, Waters) was used. The mobile phases consisting of water acidified with formic acid 0.1% (A) and acetonitrile (B), at a flow rate of 500 µL min**^−^**^1^ in gradient mode, 0–20 min: from 10% to 35% of B, 20–25 min: from 35% to 100% B. Twenty microliters were injected for each sample and three biological replicates were run for each analysis.

### 4.6. Analysis of Hormone Contents

Phytohormone analysis from vine leaves was performed following the procedure previously reported by [72], with minor modifications. Leaf samples were frozen in liquid nitrogen and homogenized. About 0.2 g was weighed into a 2 mL centrifuge tube with 1 mL of extract solution (methanol:water, 80:20, *v/v* and acidified with 0.1% acetic acid) and then shaken at 4 °C overnight in the dark. Samples were then centrifuged for 10 min at a speed of 13,000 rpm and filtered with a 0.2 μm cellulose filter. Finally, the supernatant was analyzed by HPLC-MS/MS.

The standard compounds of jasmonic acid (JA) (purity ≥95%), salicylic acid (SA) (purity ≥99%), and abscisic acid (ABA) (purity ≥98%) were supplied by Sigma-Aldrich (St Louis, MO, USA).

Instrumental analysis was performed using a 1260 Agilent Technologies system consisting of a binary pump and a vacuum degasser, connected to a Varian autosampler, Model 410 Prostar (Hansen Way, CA, USA), equipped with a 20 μL loop coupled to a Varian 310-MS TQ Mass Spectrometer. The separation of phytohormones was performed using a Luna 3 μm phenyl-hexyl (150 × 2 mm, Phenomenex, Torrance, CA, USA) under a flow rate of 200 μL min**^−^**^1^. The mobile phase consisted of a mixture of water: acetonitrile, acidified with 0.1% formic acid, and used in gradient elution mode. The chromatography run was programmed as follows: 0–7 min isocratic 40% solvent B, followed by a linear gradient from 40% to 100% B in 5 min and holding at 100% B for 4 min. The injection volume was 10 μL and the mass spectrometer was operated in the ESI (electrospray) positive ionization mode using the multiple reaction monitoring (MRM) mode. The selected quantification ion transitions were: 263 > 153 (12 eV) for ABA; 137 > 93 (16 eV) for SA and 209 > 59 (14 eV) for JA. The collision gas (Ar) pressure was set at 2 mbar for all experiments.

### 4.7. Statistical Analysis

Statistically significant differences among treatments, genotypes, or interactions were determined by performing a two-way analysis of variance (ANOVA) test. When an ANOVA test indicated that either genotype (G: ‘Nebbiolo’, ‘Moscato’) or treatment (T: CTR, AcS-Mt, K-Pho, Lam) or their interaction (G × T) was significant, the Tukey’s honestly significant difference (HSD) post-hoc test was used to separate means (*p < 0.05*) using the SPSS statistical software package (v23.0; SPSS Inc., Cary, NC, USA). Genotype (G) main effects were statistically determined by the Student’s *t*-test. The GraphPad Prism software (GraphPad Software, La Jolla, CA, USA v.6.01) was used to elaborate figure charts.

### 4.8. Accession Numbers

Raw sequences from the RNAseq libraries were deposited at the NCBI Sequence Read Archive, accession numbers from SRR8279892 to SRR8279915.

## Supplementary Materials

Supplementary Materials can be found at https://www.mdpi.com/1422-0067/21/18/6776/s1. References [73,74,75] are cited in the Supplementary Materials.
