# Supplementary material for: The Molecular Priming of Defense Responses is Differently Regulated in Grapevine Genotypes Following Elicitor Application against Powdery Mildew
Source: Int J Mol Sci. 2020 Sep 15;21(18):6776. doi: 10.3390/ijms21186776 (PMC7555711; doi:10.3390/ijms21186776)
Supplement: Supplementary file 1 [file ijms-21-06776-s001.zip › supplementary materials/Supplementary Table S1-S5_Figure S1-S2.pdf]

The molecular priming of defense responses is differently regulated in grapevine genotypes following elicitor application against powdery mildew

Chiara Pagliarani[1*], Amedeo Moine[1], Walter Chitarra[1,2], Giovanna Roberta Meloni[3,4], Simona Abbà[1], Luca Nerva[1,2], Massimo Pugliese[3,4], Maria Lodovica Gullino[3,4] and Giorgio Gambino[1]

[1]Institute for Sustainable Plant Protection, National Research Council (IPSP-CNR), Strada delle Cacce 73, 10135 Torino (Italy)

[2]Research Centre for Viticulture and Enology, Council for Agricultural Research and Economics (CREA-VE), Via XXVIII Aprile 26, 31015 Conegliano (TV), Italy

[3]Centre of Competence for the Innovation in the Agro-Environmental sector (AGROINNOVA) - University of Torino – Largo Paolo Braccini 2, 10095, Grugliasco (TO), Italy

[4]Department of Agricultural, Forest and Food Sciences (DISAFA) - University of Torino - Largo Paolo Braccini 2, 10095, Grugliasco (TO), Italy

*****Correspondence: Chiara Pagliarani
chiara.pagliarani@ipsp.cnr.it

The following Supplementary material is available for this article:

Table S1-S5*

Figures S1-S2

*due to file dimension, Table S2 and S3 are reported separately as Excel datasheets.

Table S1: **Summary statistics of RNA-sequencing libraries**. Data were obtained from leaf samples of *Vitis vinifera* 'Moscato' (MO) and 'Nebbiolo' (NE) collected from plants infected by *Erysiphe necator*. Inoculated untreated control (CTR), acibenzolar-S-methyl (AcS-Mt), potassium Phosphonate (K-Pho), and laminarin (Lam).

Sample	Total PE reads*	Total PE reads mapped§	% Mapped reads
**CTR-1_MO**	42’587’660	29'080’580	68.3
**CTR-2_MO**	45’069’190	24'223’626	53.7
**CTR-3_MO**	50'114’280	35'295’914	70.4
**AcS-Mt-1_MO**	48'493’198	34'079’924	70.3
**AcS-Mt-2_MO**	51'123’880	34'534’884	67.6
**AcS-Mt-3_MO**	54'354’606	38'405’336	70.7
**K-Pho-1_MO**	45'732’170	31'972’880	69.9
**K-Pho-2_MO**	42'315’870	29'583’148	69.9
**K-Pho-3_MO**	108'693’028	74'400’048	68.4
**Lam-1_MO**	92'930’466	65'434’592	70.4
**Lam-2_MO**	98'065’436	67'344’484	68.7
**Lam-3_MO**	87'080’686	61'017’764	70.1
**CTR-1_NE**	98'341’468	65'218’932	66.3
**CTR-2_NE**	97'011’148	68'112’540	70.2
**CTR-3_NE**	97'381’252	67'081’528	68.9
**AcS-Mt-1_NE**	81'545’010	55'142’342	67.6
**AcS-Mt-2_NE**	86'566’574	59'391’346	68.6
**AcS-Mt-3_NE**	51'844’404	29'926’484	57.7
**K-Pho-1_NE**	53'199’066	36'795’992	69.2
**K-Pho-2_NE**	52'318’438	34'671’756	66.3
**K-Pho-3_NE**	47'012’506	32'193’056	68.5
**Lam-1_NE**	48'231’786	32'621’434	67.6
**Lam-2_NE**	40'565’336	28'500’424	70.3
**Lam-3_NE**	55'072’592	37'657’256	68.4

* number of reads in which both reads of each pair end (PE) couple passed the quality check successfully
§ reads that mapped in pairs

Table S4: **Experimental outline**. Timing of treatments and pathogen (*Erysiphe necator*) inoculation in plants of *Vitis vinifera* 'Moscato' and 'Nebbiolo'. Table adapted from Nerva et al. (2019). Inoculated untreated control (CTRL), acibenzolar-S-methyl (AcS-Mt), potassium phosphonate (K-Pho), and laminarin (Lam).

**Treatments**	**August 10th 2017**	**August 17th 2017**	**August 17th 2017**	**August 26th 2017**	**September 4th 2017**	**September 5th 2017**	**September 11th 2017**	**September 19th 2017**	**September 22nd 2017**
**CTR**	-	-	*E. necator* ($1x10^5$ conidia/ml)	-	-	*E. necator* ($1x10^5$ conidia/ml)	-	-	Disease scoring and leaf sampling
**AcS-Mt (commercial formulation: Bion)**	Bion	Bion	*E. necator* ($1x10^5$ conidia/ml)	Bion	Bion	*E. necator* ($1x10^5$ conidia/ml)	Bion	Bion	
**K – Pho (commercial formulation: Century Sl)**	Century Sl	Century Sl	*E. necator* ($1x10^5$ conidia/ml)	Century Sl	Century Sl	*E. necator* ($1x10^5$ conidia/ml)	Century Sl	Century Sl	
**Lam (commercial formulation: Vacciplant)**	Vacciplant	Vacciplant	*E. necator* ($1x10^5$ conidia/ml)	Vacciplant	Vacciplant	*E. necator* ($1x10^5$ conidia/ml)	Vacciplant	Vacciplant	

Table S5: **Primer list**. Oligonucleotides used in this study for RT-qPCR analysis of candidate gene expression. For each primer pair, the gene ID (GGDB 12X V1), amplicon size (bp) and related references are reported.

Primer name	Sequence 5'-3'	Gene ID (GGDB 12X V1)	Amplicon size (bp)	Reference
VvPR1 for	ACTCATGTGTTGGTGGGCAA	VIT_03s0088g00810	108	This work
VvPR1 rev	TGACGAACCACCCTCCATTG			
VvNPF3 for	TCGTCACATCAGCACAGCTT	VIT_08s0040g00830	166	[46]
VvNPF3 rev	ATCTGCGAGCCAATGGAACA			
VvCHL for	GGCGATTATGGGTTCCATGT	VIT_07s0151g00110	70	[73]
VvCHL rev	GCCCACCTACACACTCCATCA			
VvPSBx for	TCCCCATCTCTCAAGAACTTCCT	VIT_04s0008g01730	75	[73]
VvPSBx rev	AACAGCTCCGACGATTGCA			
VvSTA for	GGCGACTCTGACTGCTTCTCA	VIT_02s0025g02790	58	[74]
VvSTA rev	CCTGGGTGCCGTTGACAT			
VvHT2 for	TACTTTTCGTGCCGGAGACC	VIT_18s0001g05570	83	[68]
VvHT2 rev	AAACCTCCGCCAGAACCAAT			
VvHT5for	AGTACGACAACCAAGGGCTACAG	VIT_05s0020g03140	60	[74]
VvHT5 rev	GAGGTCAAGCCCGCAAGATA			
VvGIN2 for	CCAACCAAGGCGATCTATG	VIT_02s0154g00090	76	[42]
VvGIN2 rev	TTGAGGCAGTGATGCTGG			
VvCwINV for	AGGAGGTGGAAAGGTTTGCATA	VIT_09s0002g02320	69	[42]
VvCwINV rev	TGGGCTTCACCGTCAATAGC			
VvOPR3 for	GTGAAGAAGAGGAAGCTCA	VIT_11s0016g01230	141	[75]
VvOPR3 rev	AGGCGACCATAGGATACC			
VvNCED for	GGTGGTGAGCCTCTGTTCCT	VIT_19s0093g00550	132	[40]
VvNCED rev	CTGTAAATTCGTGGCGTTCACT			
VvLAC14 for	ATTCGAGGCATCCAACCCTG	VIT_18s0075g00590	119	This work
VvLAC14 rev	ACATTTGAGCTTCCGGGTGT			
VvCHIT for	GGCAACTGACGCAACCATTT	VIT_04s0008g00140	82	This work
VvCHIT rev	ACATCGTGGCATGAGGGTTT			
VvMLO6 for	AGCCCACCATCTTCAATGACA	VIT_08s0040g02170	54	[59]
VvMLO6 rev	GCGTGGTGCCAGTTTCTCA			
VvUBI for	TCTGAGGCTTCGTGGTGGTA	VIT_16s0098g01190	99	[74]
VvUBI rev	AGGCGTGCATAACATTTGCG			
VvACT for	GCCCCTCGTCTGTGACAATG	VIT_04s0044g00580	100	[74]
VvACT rev	CCTTGGCCGACCCACAATA			

Figure S1: RNAseq vs RT-qPCR. Correlation between RNAseq (FPKM) and RT-qPCR results (normalized expression values) obtained from data of the expression analyses performed on transcripts (Table S5) used for validation of RNAseq results. P value reported below the equation in the graph attests the significance of the regression analysis (*P < 0.05*).

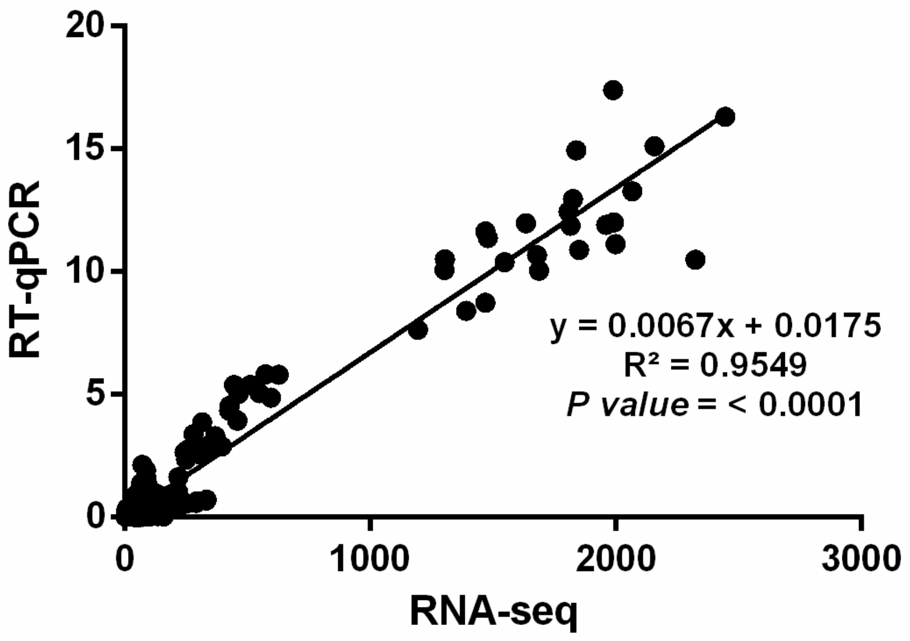

Figure S2: Additional candidate gene expression analysis. RT-qPCR expression profiles of further genes belonging to (a-b) cluster 1 (DOWN AcS-Mt, Table S3) *VvPSBX* (VIT_04s0008g01730) and *VvNPF3* (VIT_08s0040g00830), (c-d) cluster 2 (UP AcS-Mt, Table S3) *VvGIN2* (VIT_02s0154g00090) and *VvHT2* (VIT_18s0001g05570) and (e-f) cluster 3 (UP MO, Table S3) *VvLAC14* (VIT_18s0075g00590), *VvPR1* (VIT_03s0088g00700), analysed in leaf samples taken from 'Nebbiolo' and 'Moscato' plants artificially inoculated *Erysiphe necator* and then untreated (CTR) or treated with acibenzolar-S-methyl (AcS-Mt), potassium phosphonate (K-Pho), and laminarin (Lam). Ubiquitin (*VvUBI*) and Actin (*VvACT1*) genes were both used as endogenous controls for the normalization of transcript levels. Significance of genotype, treatment, and genotype × treatment (G x T) interaction was assessed by Tukey's HSD test for $P \leq 0.05$ (*), $P \leq 0.01$ (**), $P \leq 0.001$ (***) and $P \leq 0.0001$ (****) and the corresponding results are given above each graph in the figure panel; n.s. = not significant. Lower case letters above bars are reported when the G x T interaction and/or Genotype (G) main effects are statistically significant as attested by Tukey's HSD or Student's t test, respectively. Error bars represent SE. Three independent biological replicates with three technical replicates each were used for the analysis.

(following figure)

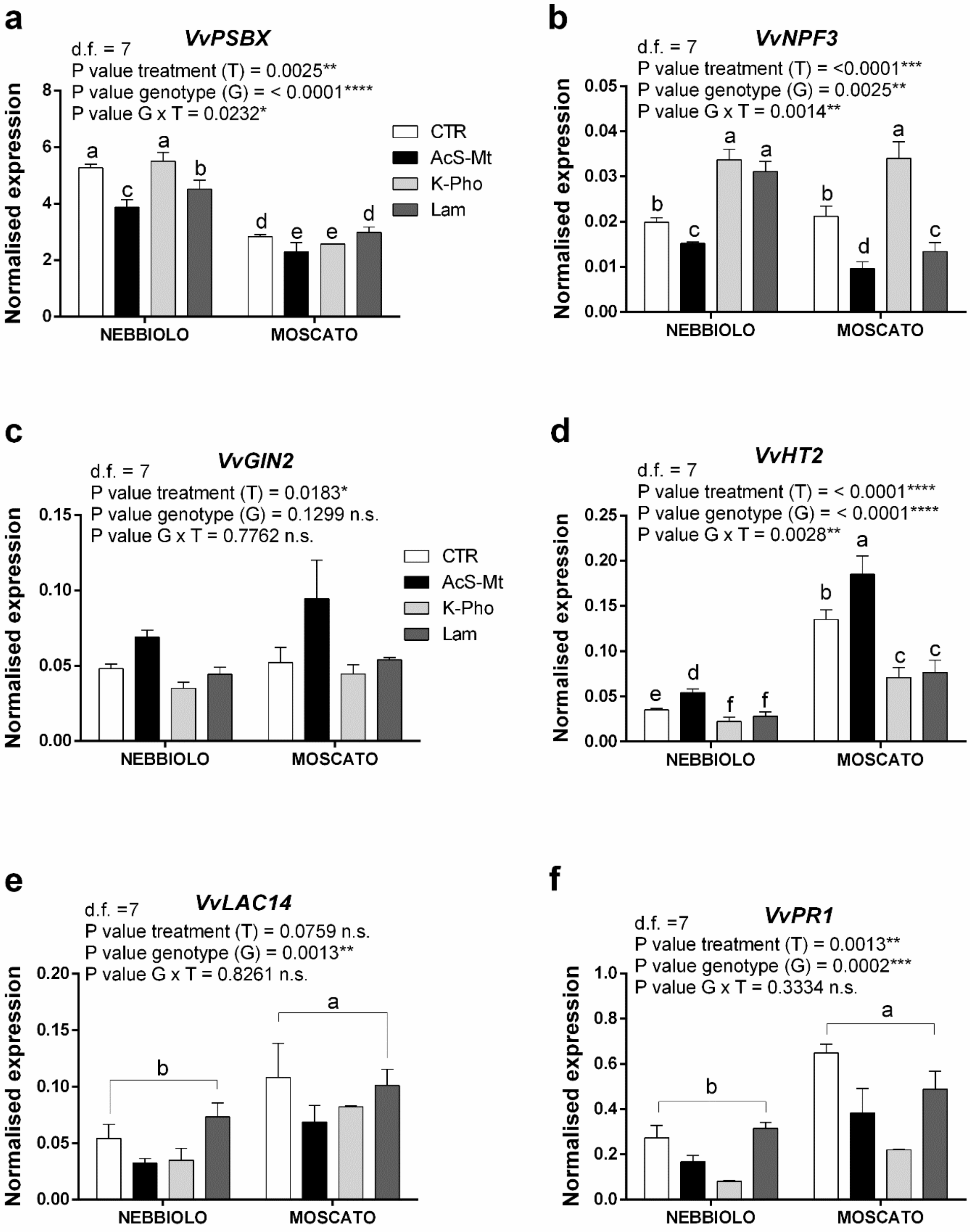
a
VvPSBX
d.f. = 7
P value treatment (T) = 0.0025**
P value genotype (G) = < 0.0001****
P value G x T = 0.0232*
Normalised expression
CTR
AcS-Mt
K-Pho
Lam
NEBBIOLO
MOSCATO
b
VvNPF3
d.f. = 7
P value treatment (T) = <0.0001***
P value genotype (G) = 0.0025**
P value G x T = 0.0014**
Normalised expression
NEBBIOLO
MOSCATO
c
VvGIN2
d.f. = 7
P value treatment (T) = 0.0183*
P value genotype (G) = 0.1299 n.s.
P value G x T = 0.7762 n.s.
Normalised expression
CTR
AcS-Mt
K-Pho
Lam
NEBBIOLO
MOSCATO
d
VvHT2
d.f. = 7
P value treatment (T) = < 0.0001****
P value genotype (G) = < 0.0001****
P value G x T = 0.0028**
Normalised expression
NEBBIOLO
MOSCATO
e
VvLAC14
d.f. =7
P value treatment (T) = 0.0759 n.s.
P value genotype (G) = 0.0013**
P value G x T = 0.8261 n.s.
Normalised expression
NEBBIOLO
MOSCATO
f
VvPR1
d.f. =7
P value treatment (T) = 0.0013**
P value genotype (G) = 0.0002***
P value G x T = 0.3334 n.s.
Normalised expression
NEBBIOLO
MOSCATO
